# Silver Diphosphine Complexes as Antimitochondrial Agents

**DOI:** 10.1155/MBD.1994.523

**Published:** 1994

**Authors:** Susan J. Berners-Price, Doreen C. Collier, Peter J. Sadler, Rodney E. Sue, David Wilkie

**Affiliations:** 1 Faculty of Science Griffith University Nathan Queensland 4111 Australia; 2 Department of Biology University College University of London Gower Street London WC1E 6BT United Kingdom; 3 Department of Chemistry Birkbeck College University of London 29 Gordon Square London WC1H 0PP United Kingdom


					SILVER DIPHOSPHINE COMPLEXES
AS ANTIMITOCHONDRIAL AGENTS
Susan J. Berners-Price,Doreen C. Collier2, Peter J. Sadler3,
Rodney E. Sue3 and David Wilkie2
Faculty of Science, Griffith University, Nathan, Queensland, Australia 4111
2 Department of Biology, University College, University of London,
Gower Street, London WC1 E 6BT, UK
3 Department of Chemistry, Birkbeck College, University of London,
29 Gordon Square, London WC1H 0PP, UK
Like their Au(I) counterparts, silver complexes of the type [M(R'2P(CH2)nPR'" 2)2 exhibit potent
cytotoxicity towards cancer cells in vitro, ,2 in addition, they show antifungal and modest antibacterial
properties.2 However, none of these has reached clinical trials because of extreme mitochondrial
toxicity related to uncoupling of oxidative phosphorylation for the gold(I) analogues.3 We now report
that the Ag(I) complex, [Ag(Et2PCH2CH2PPh2)2]NO3, (I), inhibits the growth of yeast selectively in
non-fermentable media, indicating a primary effect on mitochondrial function. Yeast cells in a
fermentable medium (containing glucose) show a high frequency of the mitochondrial mutation
petite, while toxicity (measured as cell death) is low. Aspirin in the growth medium largely reverses
the antimitochondrial effects of the complex. The chemistry of this complex and the implications for
drug design will be discussed.
We thank the Association for International Cancer Research, EC HCM programme, Royal
Society and ULIRS for their support of this work.
References
1. S.J. Bemers-Price, G.R. Girard, D.T. Hill, B.M. Sutton, P.S. Jarrett, L.F. Faucette, R.K. Johnson,
C.K. Mirabelli and P.J. Sadler, J. Med. Chem., 1990, 33, 1386-1392.
2. S.J. Bemers-Pdce, R.K. Johnson, A.J. Giovenella, L.F. Faucette, C.K. Mirabelli and P.J. Sadler, J.
Inorg. Biochem., 1988, 33, 285-295.
3, G.D. Hoke, G.F. Rush, G.E. Bossard, J.V. McArdle, B.D. Jensen and C.K. Mirabelli, J. Biol. Chem.,
1988, 263. 11203.
523

				

